# Facile decoding of quantitative signatures from magnetic nanowire arrays

**DOI:** 10.1038/s41598-020-72094-4

**Published:** 2020-09-23

**Authors:** Mohammad Reza Zamani Kouhpanji, Ali Ghoreyshi, P. B. Visscher, Bethanie J. H. Stadler

**Affiliations:** 1 Department of Electrical and Computer Engineering, University of Minnesota Twin Cities, Minneapolis, MN 55455 USA; 2 Department of Biomedical Engineering, University of Minnesota Twin Cities, Minneapolis, MN 55455 USA; 3 Seagate Technology, Bloomington, MN 55435 USA; 4 Department of Physics, University of Alabama, Tuscaloosa, AL 35487-0324 USA

**Keywords:** Magnetic properties and materials, Characterization and analytical techniques

## Abstract

Magnetic nanoparticles have been proposed as contact-free minimal-background nanobarcodes, and yet it has been difficult to rapidly and reliably decode them in an assembly. Here, high aspect ratio nanoparticles, or magnetic nanowires (MNWs), are characterized using first-order reversal curves (FORC) to investigate quantitative decoding. We have synthesized four types of nanowires (differing in diameter) that might be used for barcoding, and identified four possible “signature” functions that might be used to quickly distinguish them. To test this, we have measured the signatures of several combination samples containing two or four different MNW types, and fit them to linear combinations of the individual type signatures to determine the volume ratios of the types. We find that the signature which determines the ratios most accurately involves only the slope of each FORC at its reversal field, which requires only 2–4 data points per FORC curve, reducing the measurement time by a factor of 10 to 50 compared to measuring the full FORC.

## Introduction

Magnetic nanowires (MNWs) are increasingly impacting biomedical applications^1–6^, environmental sciences^7,8^, and quantum devices^9–12^. A unique benefit of MNWs is that they can be excited indirectly using an external field, regardless of their surroundings^5,13–15^. Especially in biomedical applications, it is essential to locate, identify, and quantify the targeted MNWs, while using multiple types of MNWs for enriching and multiplexing biological entities^4,15–17^.

MNWs have been characterized by measuring their magnetization at various applied fields using hysteresis loops and/or first-order reversal curves (FORC). Accuracy and speed are competing criteria—hysteresis loop measurements are relatively fast but contain significantly less information than FORC measurements, which typically have 50–100 × more measurement points. For example, hysteresis loops measure saturation magnetization and coercivity, which are sufficient to describe a single type of non-interacting MNW^18–21^. However, hysteresis loops cannot fully describe arrays with multiple types of MNWs, especially if there are interactions between the MNWs^22–24^. FORC, on the other hand, can separate the signals of different types of MNWs but the technique is much slower than hysteresis loops. Theoretical models, such as the mean-field model, have been used to quantify the information in FORC diagrams^25,26^ by considering perfect arrangements of MNWs with homogeneous properties. These ideal assumptions are not well-satisfied by experimental arrays of MNWs, especially those grown inside polycarbonate templates where the MNW distribution is random.

**Figure 1:**
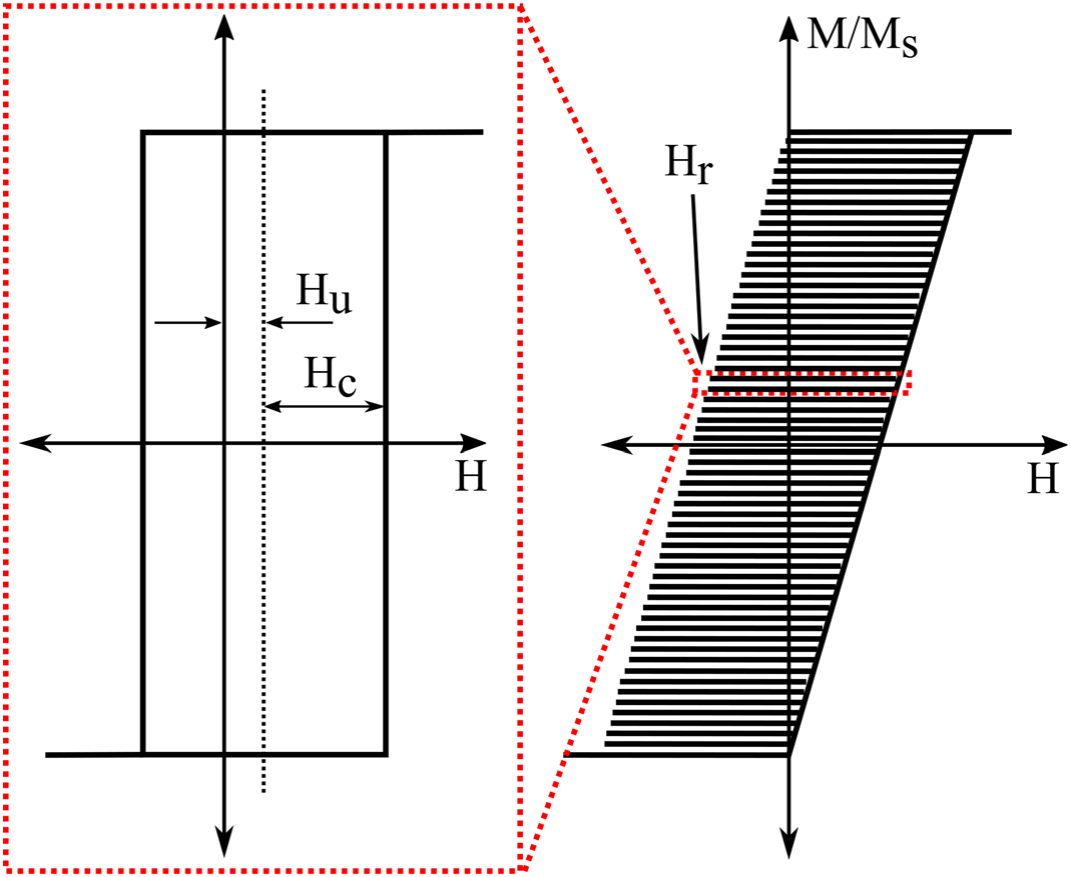
A schematic FORC measurement with interacting hysterons according to the Preisach model. The inset is a schematic of an individual hysteron, which switches down at the reversal field H_r_.

Historically, FORC measurements have provided qualitative and quantitative descriptions of complex nanomagnetic systems^21,27–29^. Mayergoyz^30,31^ proposed the current conventional FORC measurement as an identification technique for the classical Preisach model^32^, which describes magnetic hysteresis loops as a superposition of numerous independent switches, called hysterons, with rectangular hysteresis loops, such as those of isolated MNWs acting like Stoner-Wohlfarth particles^33,34^. Experimentally, FORC measurements start with applying a large magnetic field H_sat_, to ensure the positive saturation of the sample, Fig. 1. The field is then reduced to a predefined field, known as a reversal field, H_r_. The moment of the sample is measured while the applied field, H, is slowly increased back to H_sat_. This process is repeated with different reversal fields H_r_ to collect a family of magnetization curves, M(H, H_r_), as a function of H_r_ and H. Mathematically, the FORC distribution is defined^31^ as

1 $$\rho = - \frac{1}{2}\frac{{\partial^{2} M\left( {H,H_{r} } \right)}}{{\partial H\partial H_{r} }}$$

The FORC results are typically plotted as heat-maps with axes of coercivity, defined by H_c_ = (H-H_r_)/2, and interaction field, defined by H_u_ = (H + H_r_)/2, see Figures SI-1 and SI-2.

Although the FORC technique is an exceptional method for the qualitative and sometimes quantitative explanation of complex systems^36–39^, it has three main drawbacks. First, FORC usually requires very long measurement times, which is not efficient for biomedical applications or industrial quality control^40–42^. Second, smoothing is required for data processing and can induce spurious features^43–46^. Third, taking two derivatives amplifies noise that can conceal the real features.

It is our objective in this paper to use MNWs as labels or barcodes, which requires that we be able to quickly measure the amounts of each type of MNW in a combined sample. In principle this can be done by fitting the entire FORC distribution to a linear combination of single-type FORC distributions—in fact there is extensive work on this in the rock magnetism literature^27^. In that case, the components are intimately mixed crystallites of different minerals, so that interactions may invalidate the assumption of linear superposition. Our problem is a bit easier in that each type of MNW is not close to the other types in our combinations, so the pure signatures combine linearly. However, determining the entire FORC distribution is time-consuming. For our purpose, we need a signature that can be measured and fit more quickly, and that is ideally a function of one rather than two variables. Two such distributions are often extracted in FORC analysis: the coercivity distribution (P_Hc_) and interaction field distributions (P_Hu_). These are projections of the FORC heat-maps onto the H_c_ and H_u_ axes^18,24,35^ using the integrals:

2 $$P_{{H_{c} }} \left( {H_{c} } \right) = \int_{ - \infty }^{\infty } {\rho \left( {H_{c} ,H_{u} } \right)dH_{u} } \;\;{\text{and}}\;\;P_{{H_{u} }} \left( {H_{u} } \right) = \int_{0}^{\infty } {\rho \left( {H_{c} ,H_{u} } \right)dH_{c} }$$

We will use these as two of the four “signatures” for rapid characterization of our MNW systems. The last two are also functions of one variable that can be extracted from a FORC experiment (but can also be measured more rapidly): the projection the FORC heat-map onto the H_r_ axis, which is mathematically equivalent to the irreversible switching field distribution (ISFD), and the backfield remanence magnetization (BRM), both of which will be defined below.

## Experimental methods

As a proof of concept, four types of cobalt (Co) magnetic nanowires (MNWs) with average diameters of 32 nm, 55 nm, 110 nm, and 208 nm were prepared using a well-established template-assisted electrodeposition technique^20,21^ (see SI including Figure SI-3). Each of the four MNW types was measured individually with the FORC technique (magnetic field applied parallel to the MNWs axes), Figure SI-1. Next, several combinations were created with at least two different types of MNWs, and the FORC measurements were repeated, Figure SI-2. For quantitative decoding, the individual magnetic signatures were extracted from each combination FORC measurement using four different signatures: (1) the coercivity distribution (P_Hc_), (2) the interaction field distribution (P_Hu_), (3) irreversible switching field distribution (ISFD), and (4) the backfield remanence magnetization (BRM).

The first and second signatures (P_Hc_ and P_Hu_) are calculated using Eq. (2) which project the FORC heat-maps on the H_c_ and H_u_ axes, respectively. The third signature is related to the “switching field distribution” (SFD) which is conventionally defined as the derivative of the upper branch of the hysteresis loop [in our notation, M(H, H_r_) at H = H_r_]:

3 $${\text{SFD}}\left( H \right) = \frac{1}{2} \frac{{\partial M\left( {H,H} \right)}}{\partial H} = \frac{1}{2}\left. {\frac{{\partial M\left( {H,H_{r} } \right)}}{{\partial H_{r} }}} \right|_{{H_{r} = H}} + \frac{1}{2}\left. {\frac{{\partial M\left( {H,H_{r} } \right)}}{\partial H}} \right|_{{H_{r} = H}}$$

**Figure 2:**
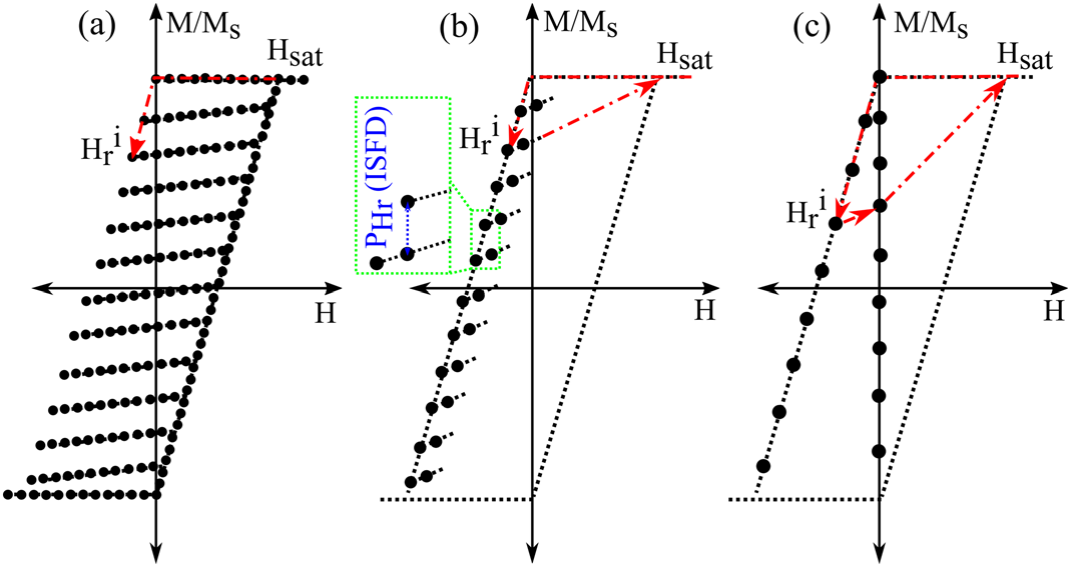
Schematic comparison of conventional FORC and alternative methods with necessary data (black dots). The red dashed lines show the direction of the field change. (**a**) Conventional FORC typically measures ~ 100 data points for each reversal curve, (**b**) ISFD requires only two data points on each reversal curve (as shown), and (**c**) BRM measures magnetization at zero field after the application of each H_r_.

The term “switching field distribution” is slightly unfortunate because elements “switch” at specific fields only in a system of ideal Preisach hysterons—however, the term is well established so we will use it here. Note that the change in M upon switching is twice the saturation moment—if we define SFD to be the amount of saturation moment switching per unit field, we get the factor of 1/2 shown in Eq. (3). The first term in Eq. (3) vanishes for a reversible system, so we will refer to it as the irreversible SFD (ISFD), and the second term as the reversible SFD. It is schematically shown as the blue line in Fig. 2b. Mathematically, ISFD is also proportional to the projection of the FORC heat-maps onto the H_r_ axis by integrating over all applied fields:

4 $$\int_{{H_{r} }}^{\infty } {\rho \left( {H,H_{r} } \right)dH} = - \frac{1}{2}\left. {\frac{{\partial M\left( {H,H_{r} } \right)}}{{\partial H_{r} }}} \right|_{H = \infty } + \frac{1}{2}\left. {\frac{{\partial M\left( {H,H_{r} } \right)}}{{\partial H_{r} }}} \right|_{{H = H_{r} }} = 0 + \frac{1}{2}\left. {\frac{{\partial M\left( {H,H_{r} } \right)}}{{\partial H_{r} }}} \right|_{{H = H_{r} }}$$

Note that the first term is zero because the magnetization does not change with H_r_ at large values of applied field (H) due to saturation.

Finally, the fourth signature (BRM) is the value of magnetization of the sample when the applied field is brought to zero after each application of different H_r_ values, Fig. 2c.

To determine which of these four magnetic signatures reliably and quantitatively decodes the amounts and types of the MNW in the combinations, we fit the signature of the combination to a linear superposition of the magnetic signatures from individual MNWs types (“volumetric fit” in the figures). For example, for the P_Hc_ signature of a combination of MNWs with diameters of 55 nm and 208 nm,

5 $$Volumetric\,fit\,for\,P_{Hc} = \alpha_{{55\,{\text{nm}}}} P_{{Hc55\,{\text{nm}}}} + \alpha_{{208\,{\text{nm}}}} P_{{Hc208\,{\text{nm}}}}$$

where the volume fraction *α*_55nm_ = (volume of Co in 55 nm wires)/(total volume of Co) and similarly for *α*_208 nm_, so that *α*_55 nm_ + *α*_208 nm_ = 1. The RMS error is defined as

6 $$RMS = \sqrt {\frac{1}{N}\mathop \sum \limits_{i = 1}^{N} \left( {Exp. data - Volumetric\,fit} \right)^{2} }$$

where *N* is the number of data-points for the corresponding signature. The *α*_*i*_ coefficients are found by minimizing the RMS. The volume ratio (x) is then

7 $$x = \frac{{\alpha_{{208\,{\text{nm}}}} }}{{\alpha_{{55\,{\text{nm}}}} }}$$

Each magnetic signature is explained in detail below, and then we explain how the minimum number of points shown in Fig. 2b can be used to obtain the best quantitative signatures with orders of magnitude fewer data points than conventional FORC.

## Results

The FORC data and heat-maps were measured and processed using the conventional FORC protocols to obtain signatures for the four Co MNWs samples that contained a single type of MNW (32 nm, 55 nm, 110 nm, and 208 nm), Figure SI-1. Similar conventional FORC measurements and processing were performed for all six combinations of two MNW types and for one combination that included all four MNW types, Figure SI-2.

**Figure 3:**
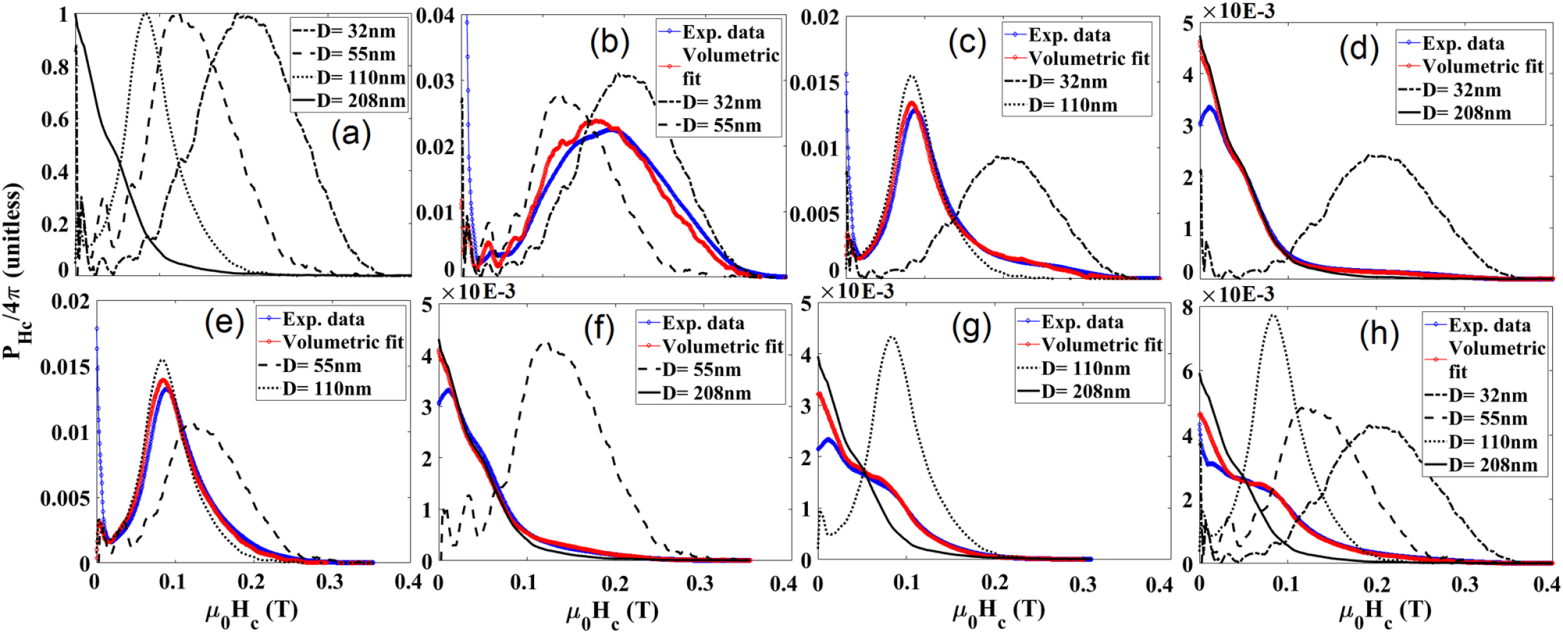
Coercivity distribution (P_Hc_, determined by taking an integral over H_u_ from FORC heat-maps) for (**a**) normalized for individual types of MNWs, (**b**–**g**) different combinations of two types of MNWs, as indicated in the legend and (**h**) one combination of four types. The blue lines show measurements of combinations, and the red lines show the best match for combinations of the individual signatures (from **a**) using volume ratio, see Table 1.

**Figure 4:**
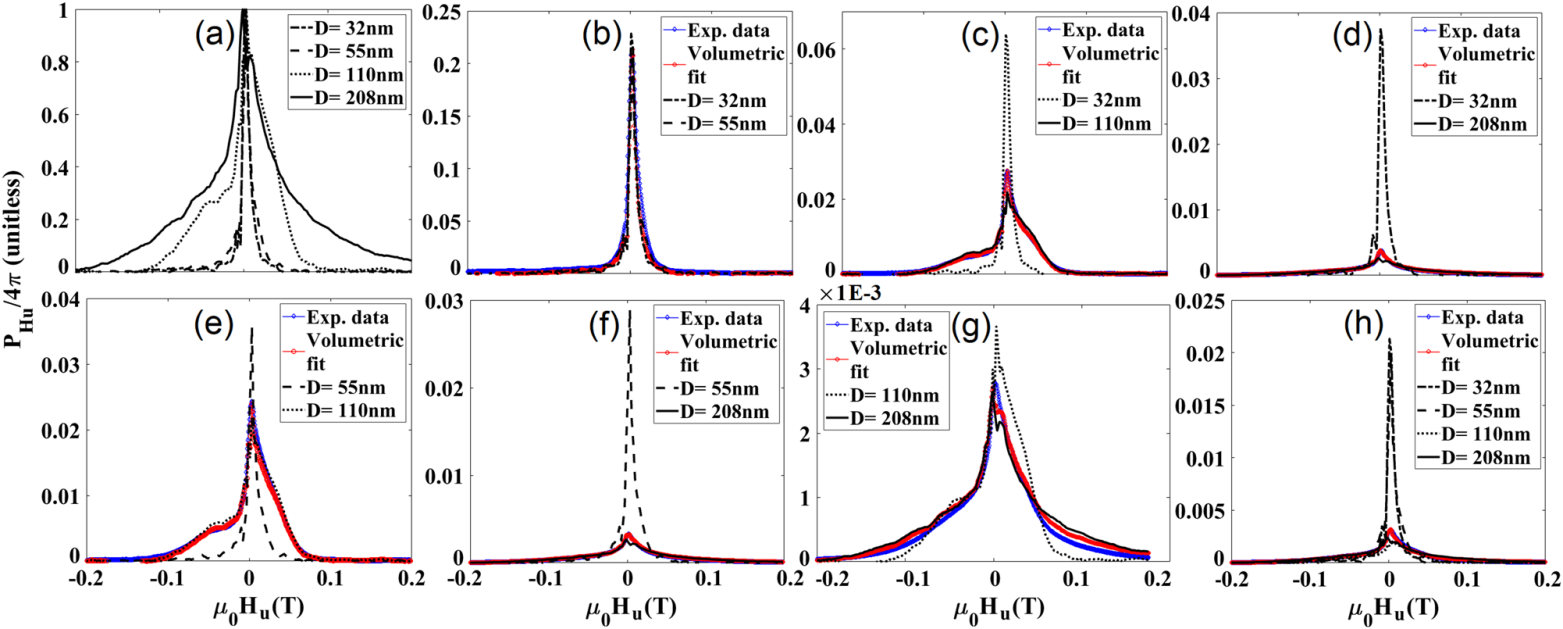
Interaction distribution (P_Hu_, the integral over H_c_ from the FORC heat-maps) for (**a**) normalized for individual types of MNWs, (**b**–**g**) different combinations of two types of MNWs, as indicated in the legend and (**h**) one combination of four types . The blue lines show measurements of combinations, and the red lines show the best match for combinations of the individual signatures (from **a**) using volume ratio, see Table 1.

For quantitative decoding, the FORC heat-maps were first projected on the H_c_ and H_u_ axes to find the coercivity distribution (P_Hc_) and interaction distribution (P_Hu_), respectively. The P_Hc_ and P_Hu_ signatures of individual types of nanowires are shown in parts (a) of Figs. 3 and 4. Combinations of these individual types were then measured (blue lines) and the volume fractions *α*_*i*_ were chosen to minimize the RMS error (Eq. 6). Table 1 gives the resulting relative volume fractions.

**Table 1:** Comparison of extracted volume ratios. The ratio x is normalized to the first type listed, i.e., in the first row *x* = *α*_55 nm_/*α*_32 nm_.

	1:x	x from P_Hc_		x from P_Hu_		x from ISFD		x from BRM	
	Known x	Fit	Error	Fit	Error	Fit	Error	Fit	Error
**Combinations of two**									
32 nm:55 nm	1.1	0.38	− 0.63	2.6	1.5	0.70	− 0.33	0.47	− 0.56
32 nm:110 nm	5.8	42	6.3	5.1	− 0.11	2.6	− 0.54	21	2.7
32 nm:208 nm	23	880	37	23	− 0.01	20	− 0.13	120	4.0
55 nm:110 nm	5.5	27	3.8	4.5	− 0.18	2.3	− 0.58	12	1.2
55 nm:208 nm	22	440	19	34	0.52	20	− 0.09	98	3.4
110 nm:208 nm	4.0	11	1.8	14	2.5	3.4	− 0.15	3.5	− 0.14
**Combination of four**									
32 nm	1	1	0	0	–	1	0	1	0
55 nm	1.1	0.95	− 0.14	0.091	− 0.92	1.2	0.12	0.27	− 0.75
110 nm	5.8	26	3.4	0.73	− 0.87	2.8	− 0.52	4.2	− 0.27
208 nm	23	270	11	16	− 0.30	16	− 0.32	11	− 0.53

Of our first two signatures, the projections onto the H_u_ and H_c_ axes, P_Hu_ is the more effective signature for most combinations with the largest error being 50% overestimation of 208 nm MNWs in a combination with 55 nm MNWs. This may seem like a large error, but many multiplexed nanoparticle diagnoses to date use log scales to plot populations due to large potential offsets^5^. For example, fluorescence labels have large errors due to background lighting and bleaching^4^. While this is the case, here we are interested in finding the best magnetic signature for decoding MNWs, so that these signatures are known for comparisons to other labelling systems in future studies.

The first two signatures P_Hu_ and P_Hc_ require taking two derivatives of the FORC data followed by an integral, but this can be avoided by using the irreversible switching field distribution (ISFD) or backfield remanence magnetization (BRM). Both ISFD and BRM can be obtained directly from the raw data (see Fig. 2). Specifically, ISFD is the change in magnetization at the same applied field when we change the reversal field, Fig. 2b. BRM is the magnetization at zero applied field after applying and removing each H_r_, Fig. 2c.

**Figure 5:**
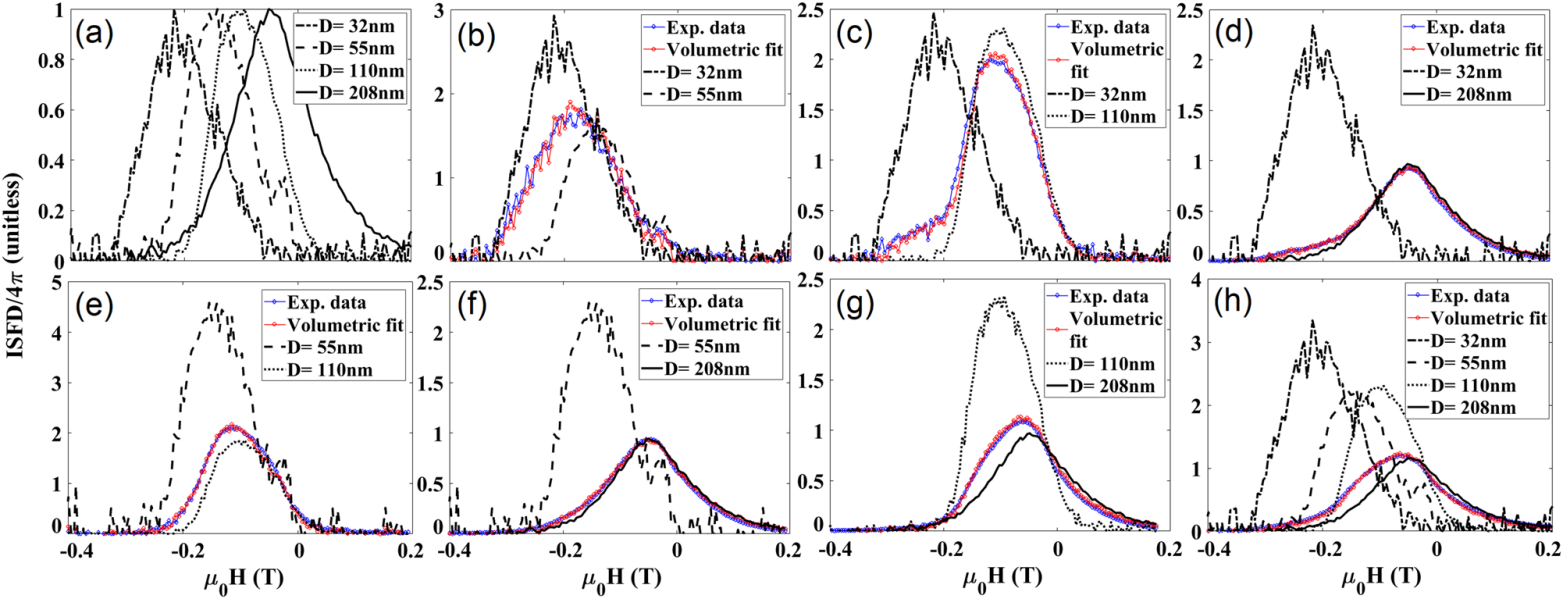
Irreversible switching field distribution (ISFD) results, (**a**) normalized for individual types of MNWs, (**b**–**g**) different combinations of two types of MNWs, as indicated in the legend and (**h**) one combination of four types. The blue circles show measurements of combinations, and the red circles show the best match for combinations of the individual signatures (from **a**) using volume ratio, see Table 1.

ISFD as a signature can be characterized by two parameters: (I) the amplitude of the local peak associated with each MNW type, proportional to the volume fraction of that type), and (II) the relative location of the peaks (dominated by the coercivity of the MNW type), Fig. 5a. For combined samples, ISFD broadens and forms two local peaks associated with the coercivities of the individual MNWs in each combination where the heights of the peaks indicate the amount of each MNW relative to another.

It should be emphasized that the sensitivity of ISFD depends on both of the amount, the fraction of irreversible switching, and coercivity of component MNWs, with the latter being the dominant parameter. Therefore, combinations of MNWs can be designed for optimal quantitative decoding by combining high coercivity and low coercivity “signatures”^15^. For example, in Fig. 5d, the combination contains 22 × more volume of 208 nm-diameter MNWs than 32 nm-diameter MNWs. However, this ratio was easily quantified with high accuracy due to separated peaks in ISFD, third column parameter in Table 1. Combinations of 32 nm or 55 nm MNWs with 110 nm MNWs had the most error (~ 50%) due to the combination of large volume ratios (~ 1:5) and the similarities in coercivity. When the volume ratio was closer (1:1 or 1:4), the coercivities of MNW “barcodes” could be closer without as much impact on decoding the combination. This technique also yielded the best results for decoding all four Co MNW codes from the single 4-way combination, lower section of Table 1.

**Figure 6:**
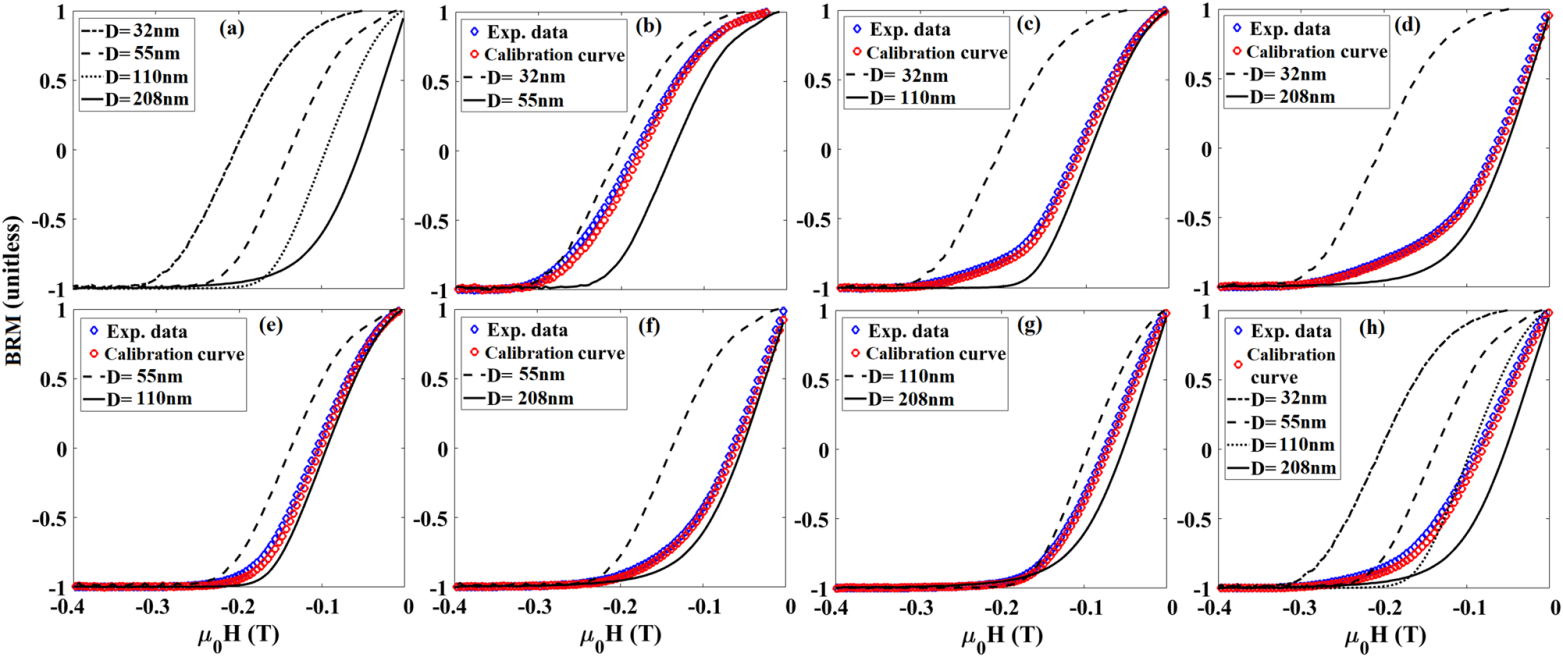
Normalized backfield remanence magnetization (BRM) results for (**a**) individual types of MNW, (**b**–**g**) different combinations of two types of MNWs, as indicated in the legend and (**h**) one combination of four types. The blue circles show measurements of combinations, and the red circles show the best match for combinations of the individual signatures (from **a**) using volume ratio, see Table 1.

BRM curves for single MNW types and combinations are shown in Fig. 6. Since the combinations had different magnetic moments, we normalized BRM with respect to their saturation backfield remanence (remanence of the major hysteresis loop, M_sbr_) to render them from − 1 to + 1. From Fig. 6, it can be seen that the BRM value of any combination is always between the BRM values of the individual MNWs in the combination. Therefore, the BRM shift in the combinations determines the amounts of each MNW present. Two features characterize BRM as a signature^47–50^: (I) the field where it is zero, which is average coercivity of the MNWs, and (II) its overall slope which is correlated to the interaction fields. The fourth parameter in Table 1 has the results of volume ratio calculations. Although this measurement can be very fast compared to P_Hc_ and P_Hu_, ISFD appears to be the best signature for these Co MNWs.

As mentioned in the Experimental Method section, the volume ratio of MNWs present in each combination was calculated using a weighted sum of the individual signatures (parts (a) of the preceding figures) where RMS error between each calculated curve and its respective measured combination curve was minimized. The ratios of these weights, which are the volume of each MNW type, determine to the volume ratios of the MNWs present in each combination. For each of the four magnetic signatures (P_Hc_, P_Hu_, ISFD, and BRM), these volume ratios were tabulated with the known volume ratio for comparison in Table 1. Italic emphasis highlighting was used to show which MNW combinations-signature pairs measured the volume ratio within a factor of 2 (corresponding to + /− 100% error). Most commonly used nanobarcodes, especially in the nanomedicine or biology, use optical nanobarcodes such as fluorescent dyes or quantum dots nanoparticles^4,5,13,15,51^. These barcode methods typically plot calculated values and errors on log scales, where a factor of 2 is quite small. In this regard, ISFD appears to be an excellent signature for overall decoding MNW combinations. The most effective multiplexing systems, however, will use a combination of techniques. For example, fluorescently-labelled magnetic nanowires could be detected by independent optical and magnetic techniques. Here, we simply report a promising magnetic technique, which has great potential to help future studies of many kinds.

**Figure 7:**
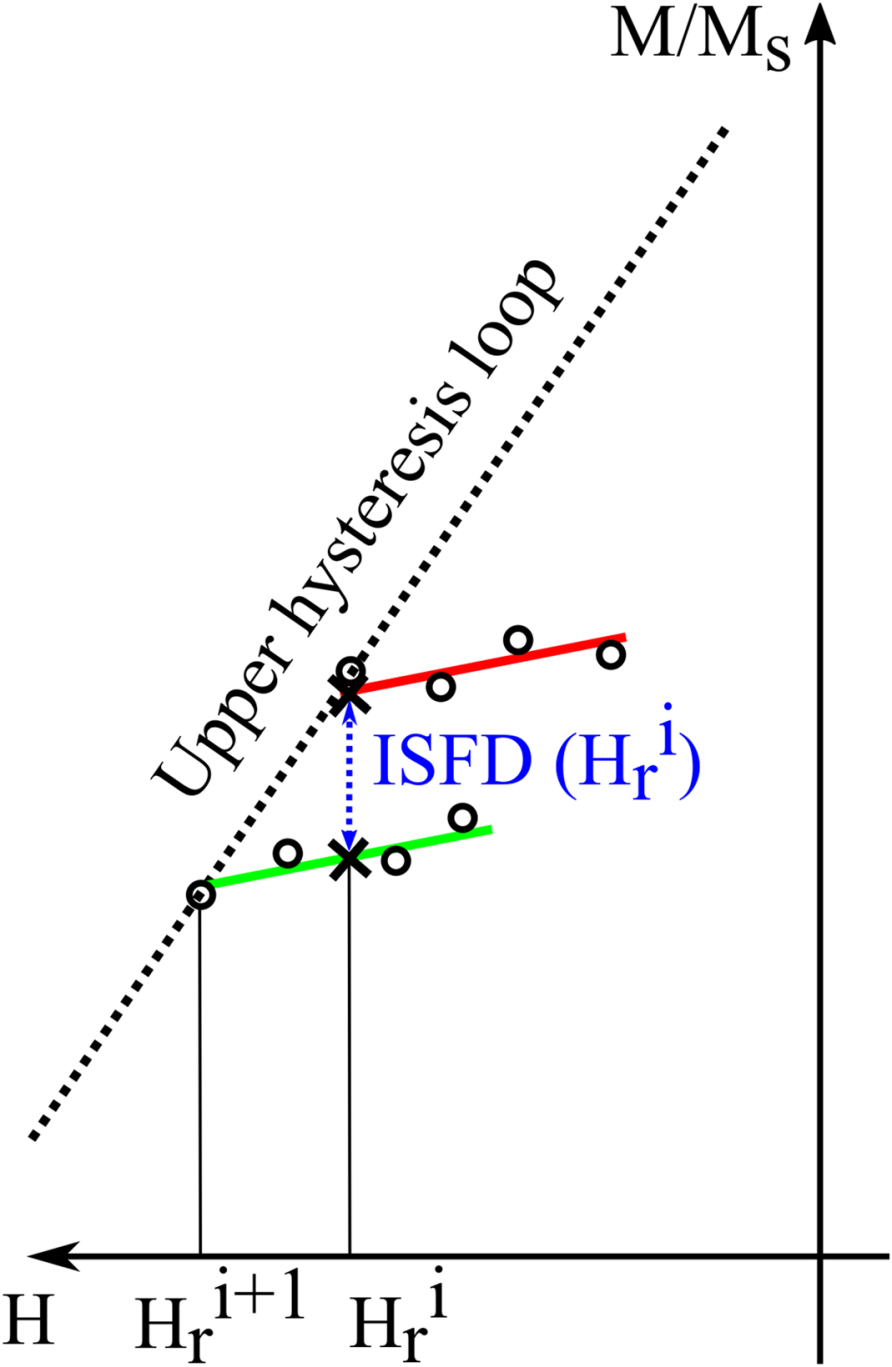
Calculation of smoothed ISFD: The upper (red) FORC begins at H_r_^i^ and has four measured points (black dots). A straight red line is least-squares fit to these points. The lower FORC begins at H_r_^i+1^ and its points are similarly fit to the green line. The black Xs are the points on the two lines with H = H_r_^i^, and the vertical distance between them is proportional to the ISFD.

As alluded to above, both ISFD and BRM can also be measured much more quickly than conventional FORC, and involve much simpler and faster data processing. This makes ISFD even more ideal for real-time diagnosis and quality control. Specifically, these signatures do not require massive data processing as required by the conventional FORC analysis^43–46^. For example, conventional FORC analysis typically requires 20–100 curves with 20–100 points each (= 400 to 10,000 points). In contrast, Figs. 2b and 7 show how the ISFD can be calculated from substantially fewer points on each magnetization curve (= 40–200 points). The results in Fig. 5 were calculated using only two points—to test the effect of smoothing, we repeated the analysis using 4 points (as shown in Fig. 7) and similarly with 6 points. The results are given in Figures SI-5 and SI-6, and there was not a notable deviation in ISFD.

Also, BRM only requires the magnetization at one point, namely zero-field (H = 0) after applying each reversal field (= 20–100 points). It should be emphasized that the BRM measurement is slightly different from the standard remanence measurements, such as isothermal remanence or DC demagnetization remanence, for example^50,52–55^. The BRM protocol saturates combinations before applying and removing each reversal field while the standard remanence protocols measure the remanence after applying and removing a continuous ascending or descending field. Although this signature was not highly effective for the Co MNWs in this study, future magnetic nanoparticles could be engineered to have large BRM differentiation to take advantage of this potentially fast decoding method.

## Conclusions

Irreversible switching field distribution (ISFD) was identified as an overall promising signature for decoding MNWs. Both ISFD and BRM can be determined 10–50 × faster than conventional FORC signatures with much simpler data processing. Regardless of the type of MNW, measurement instrument, and the data acquisition system, ISFD and BRM are fast because they require substantially fewer data points. In summary, ISFD has great potential to accelerate decoding, enabling new industry-friendly quality control and real-time diagnosis.

## Supplementary information

Supplementary file1

## Data Availability

The datasets collected and/or analyzed during the current study are available from the corresponding author on a reasonable request.
