# Supplementary material for: Facile decoding of quantitative signatures from magnetic nanowire arrays
Source: Sci Rep. 2020 Sep 23;10:15482. doi: 10.1038/s41598-020-72094-4 (PMC7512014; doi:10.1038/s41598-020-72094-4)
Supplement: Supplementary file 1 — Supplementary file1 [file 41598_2020_72094_MOESM1_ESM.pdf]

Supplementary information (SI) for:

Facile decoding of quantitative signatures from magnetic nanowire arrays

Mohammad Reza Zamani Kouhpanji[1,2], Ali Ghoreyshi[3], P.B. Visscher,[4] Bethanie J. H. Stadler[1,*]
[1]Department of Electrical and Computer Engineering, University of Minnesota Twin Cities, MN 55455 USA; [2]Department of Biomedical Engineering, University of Minnesota Twin Cities, MN 55455 USA; [3]Seagate Technology, Bloomington, MN 55435 USA; [4]Department of Physics and Astronomy, University of Alabama Tuscaloosa Alabama, 35487-0234 USA.
* Corresponding author, Email: stadler@umn.edu, Tel.: +1(612) 626-1268.

FORC measurements

The magnetic measurements were done using MicroMag Vibrating Sample Magnetometer (μVSM), Princeton Measurements Corporation, at room temperature. To determine the field ranges for our FORC measurements, we first made a hysteresis loop measurement. The FORC range is specified by giving minimum and maximum interaction fields, which were chosen as the negative and positive half of the anisotropy field, respectively. The coercivity field range was selected from zero to the anisotropy field. The saturation field was set at 1.0 T. Depending on the magnetic strength of the samples and the overall shape of their hysteresis loop, the number of FORC curves and the averaging time were selected. For example, since the overall shape of the 30nm sample's hysteresis loop was showing almost no interactions between the nanowires (meaning the FORC distribution is confined to the horizontal axis), we chose larger FORC number (smaller field steps) to capture all details. The data was processed using the FORCinel software package with the optimum smoothing factors predicted by the software. Figures SI-1 and SI-2 show the FORC measurements and heat-maps for the individual samples.

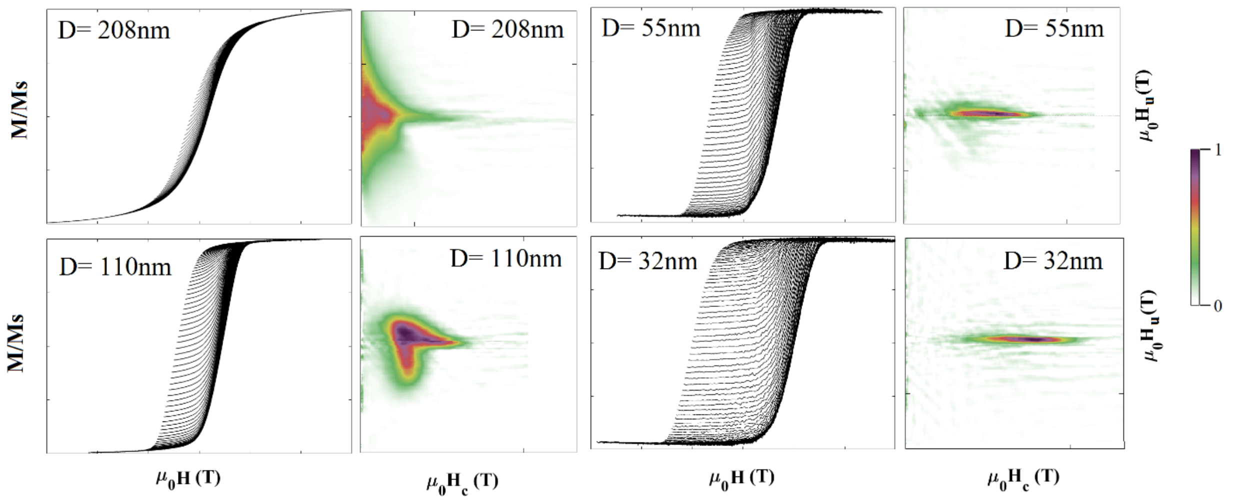

Figure SI-1: FORC measurements and heat-maps for single diameter MNW calibration samples. FORC was normalized to have a common color legend between the samples.

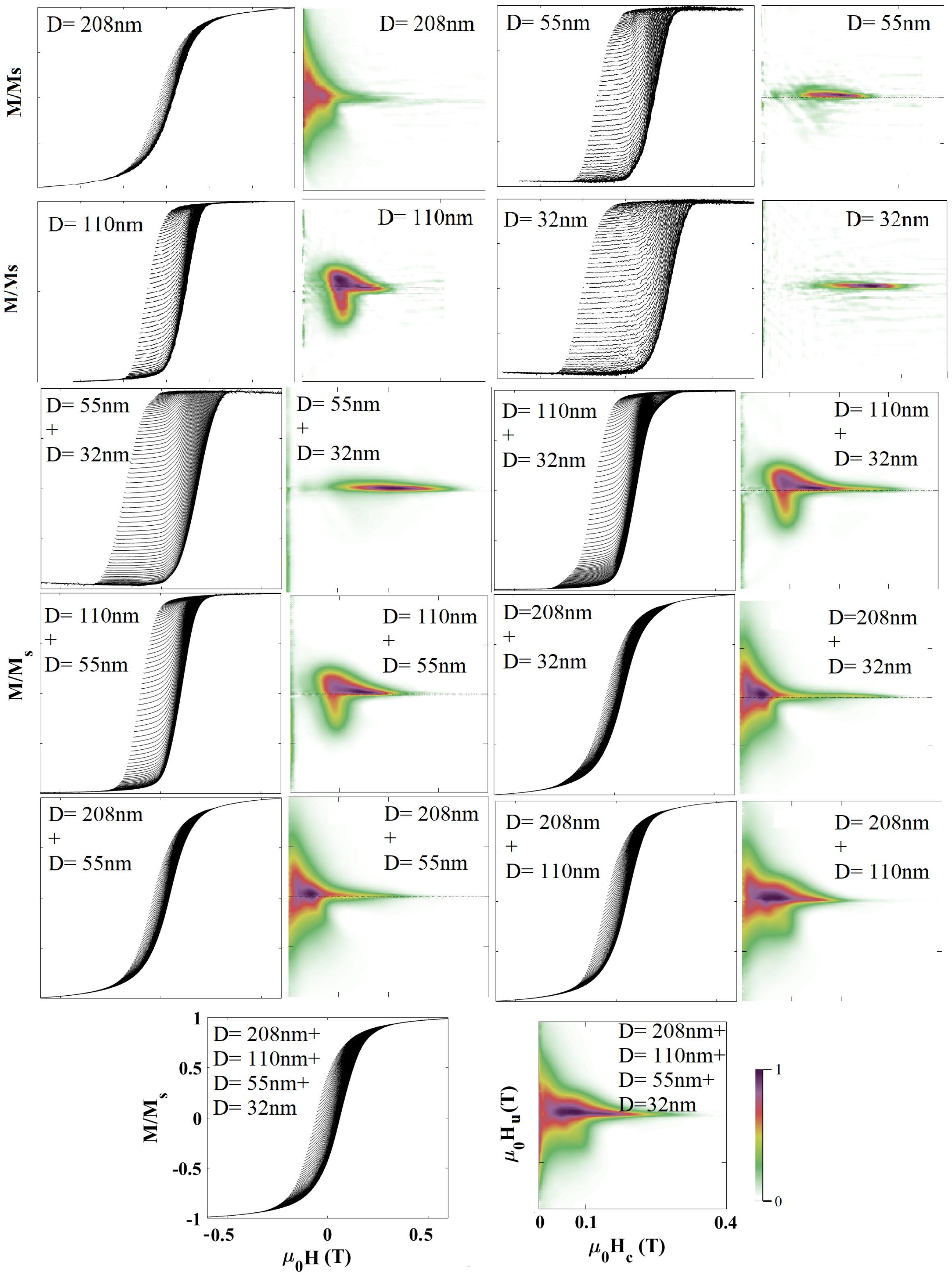

Figure SI-2: FORC measurements and heat-maps for mixed samples.

Magnetic nanowire (MNW) electrodeposition

Cobalt (Co) MNWs were electrodeposited into track-etched polycarbonate templates with a broad range of fill factors (the cross-section ratio of the MNWs to the total surface of the template = 0.5% - 12%). Figure SI-3 shows the SEM images of track-etched polycarbonate templates and the MNWs.

First, a 50nm layer of chromium (Cr) was evaporated on one side of the templates as the adhesion layer for the conductive layer, which was a 500nm copper (Cu) film. The templates were placed as the cathode in a three-electrode electrodeposition system, where a platinum (Pt) mesh was the counter electrode and a standard Ag/AgCl electrode was used as the reference electrode. The electrolyte consisted of 0.5M boric acid and 0.9M cobalt sulfate, where the pH was adjusted at 6.5 using sodium hydroxide. The electrodeposition was conducted by applying a constant voltage of 1V.

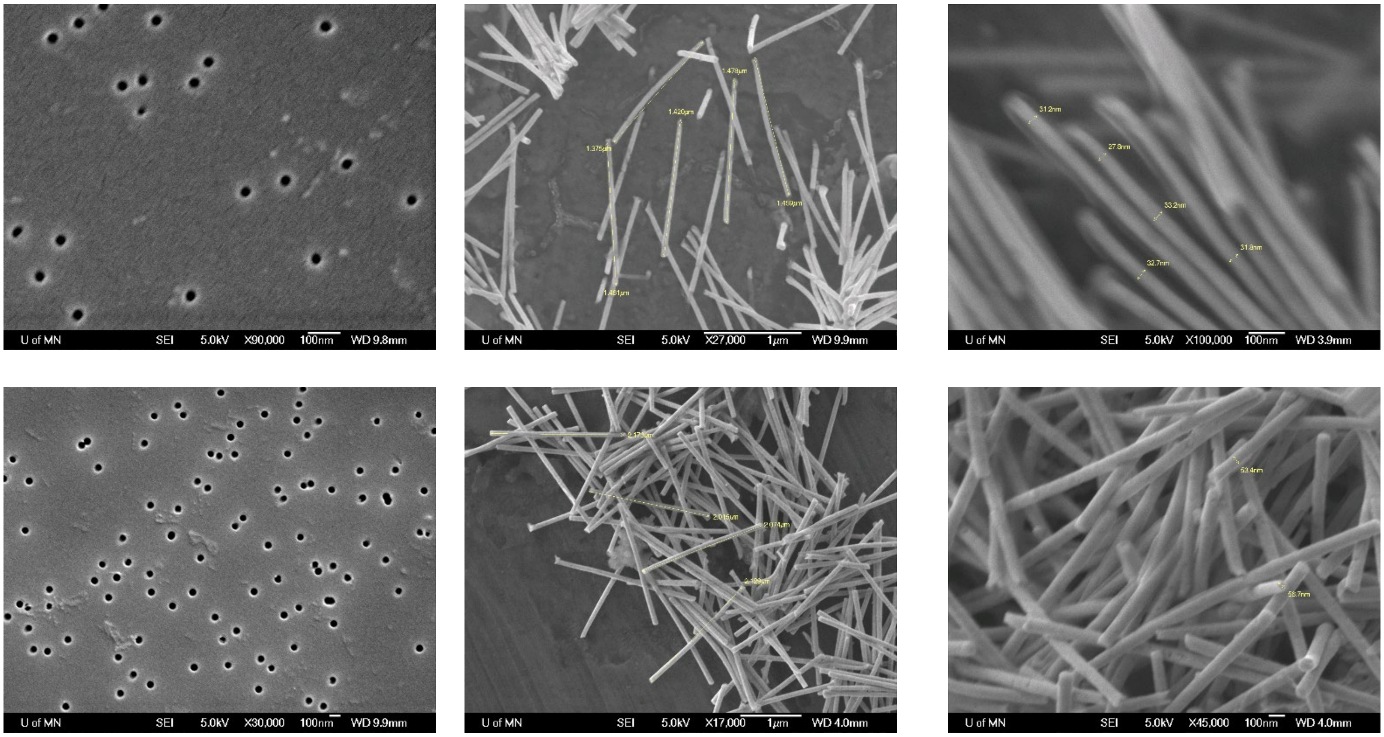

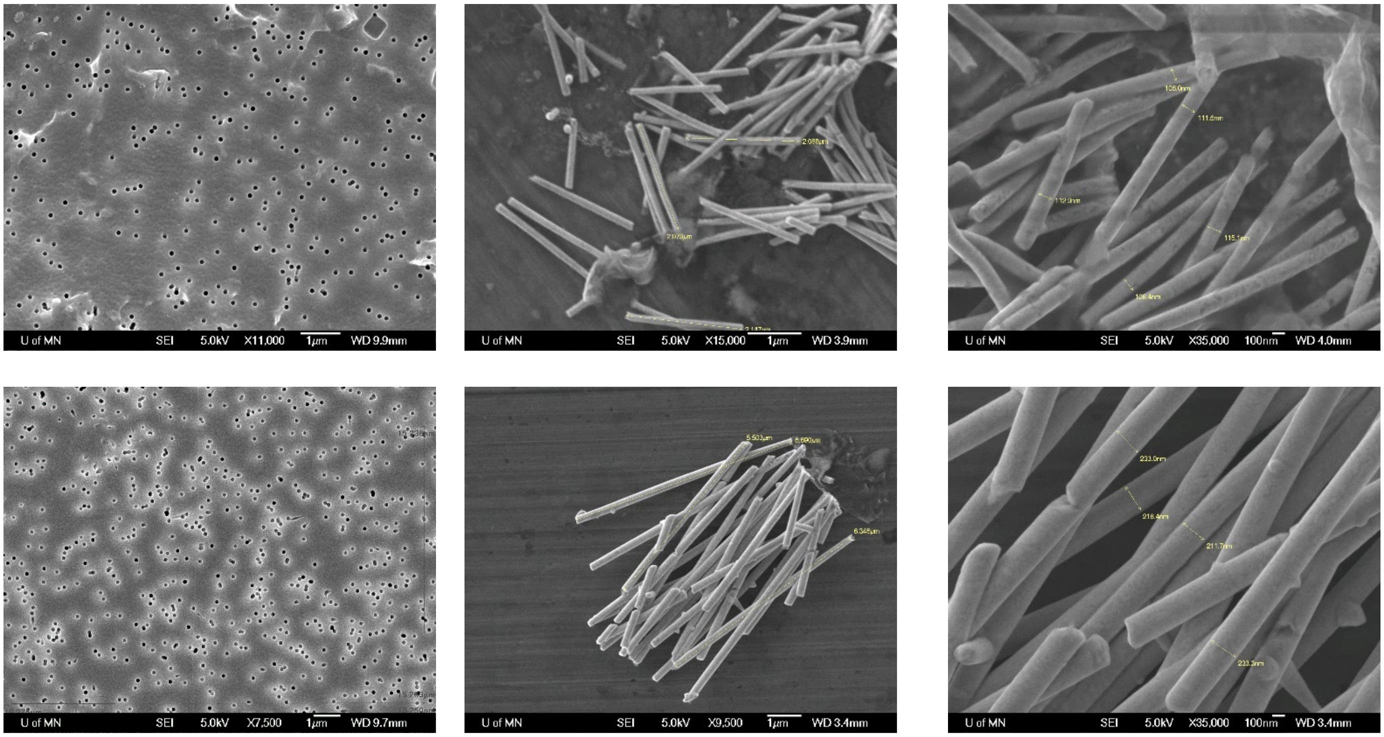

Figure SI-3: Typical SEM images of the polycarbonate templates and the MNWs. The rows contain images of 32nm, 55nm, 110nm, and 208nm diameter nanowires, respectively.

Proposed measurements

The ISFD measurement starts by applying a large field to ensure the saturation of the sample. The field then is reduced the field to a reversal field. Then the field returns back one step to the same field of the previous reversal field while measuring the magnetization. Then the field should jump back to the saturation filed at one step to repeat the same process for the next reversal fields. The solid black lines in Figure SI-4 shows the data collected in this method, here we collected 5 data points from the reversal field for better visualization. Similarly, the protocol for the backfield remanence magnetization (BRM) starts by applying a large field to ensure the saturation of the sample. The field then is reduced the field to a reversal field. Then the field goes to zero at one step to

measure the magnetization. Afterward, the field jumps back to the saturation filed at one step to repeat the same process for the next reversal fields.

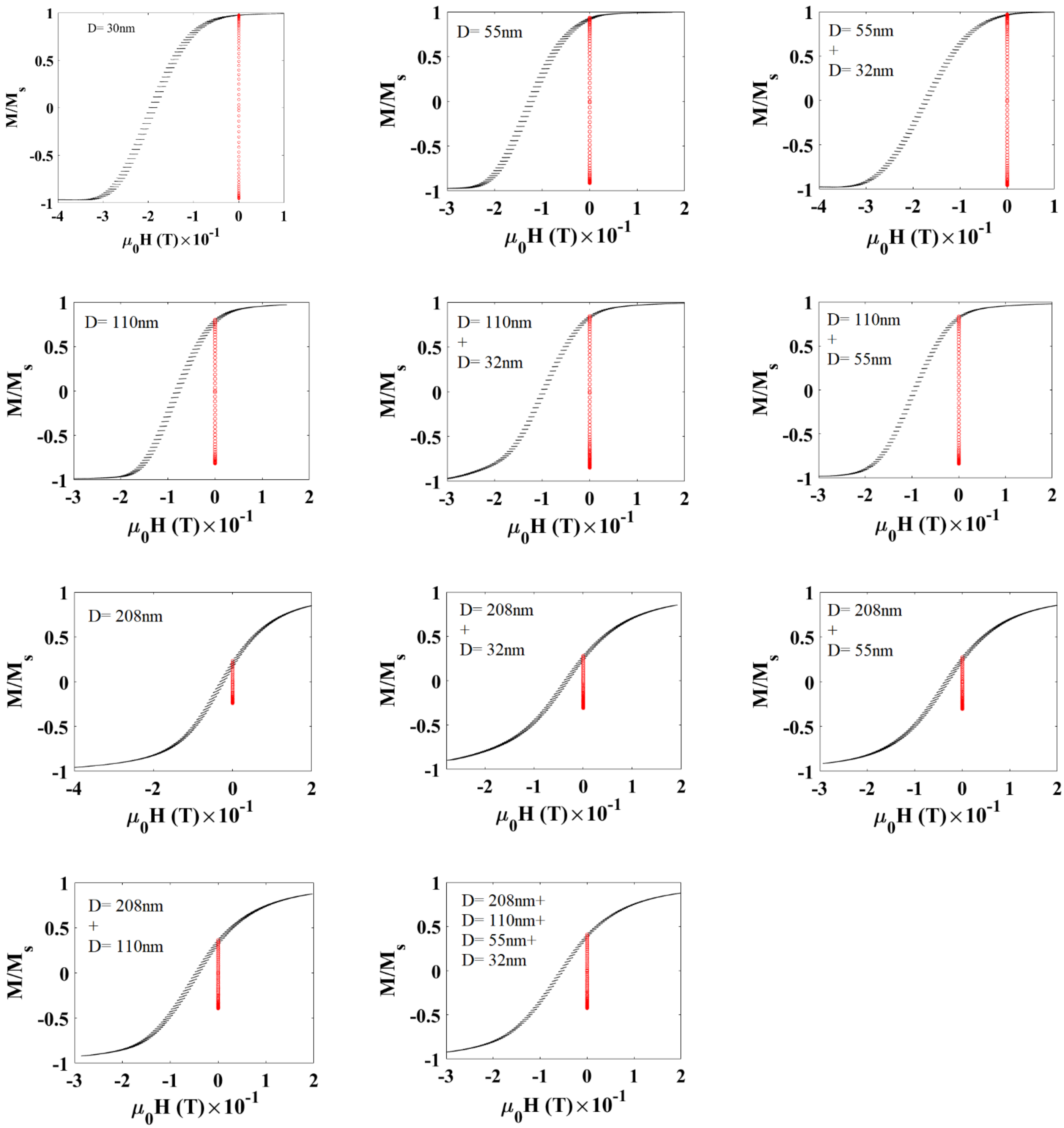

Figure SI-4: showing the data in the context of the proposed measurements. The black solid lines are the data for calculating the ISFD, and the red dots are for calculating the BRM.

In Figure SI-5, we examine the number of the data point's effects on the ISFD to evaluate the robustness of the proposed measurements. As can be seen, the deviation for the ISFD is not significant, and 2 data points are sufficient.

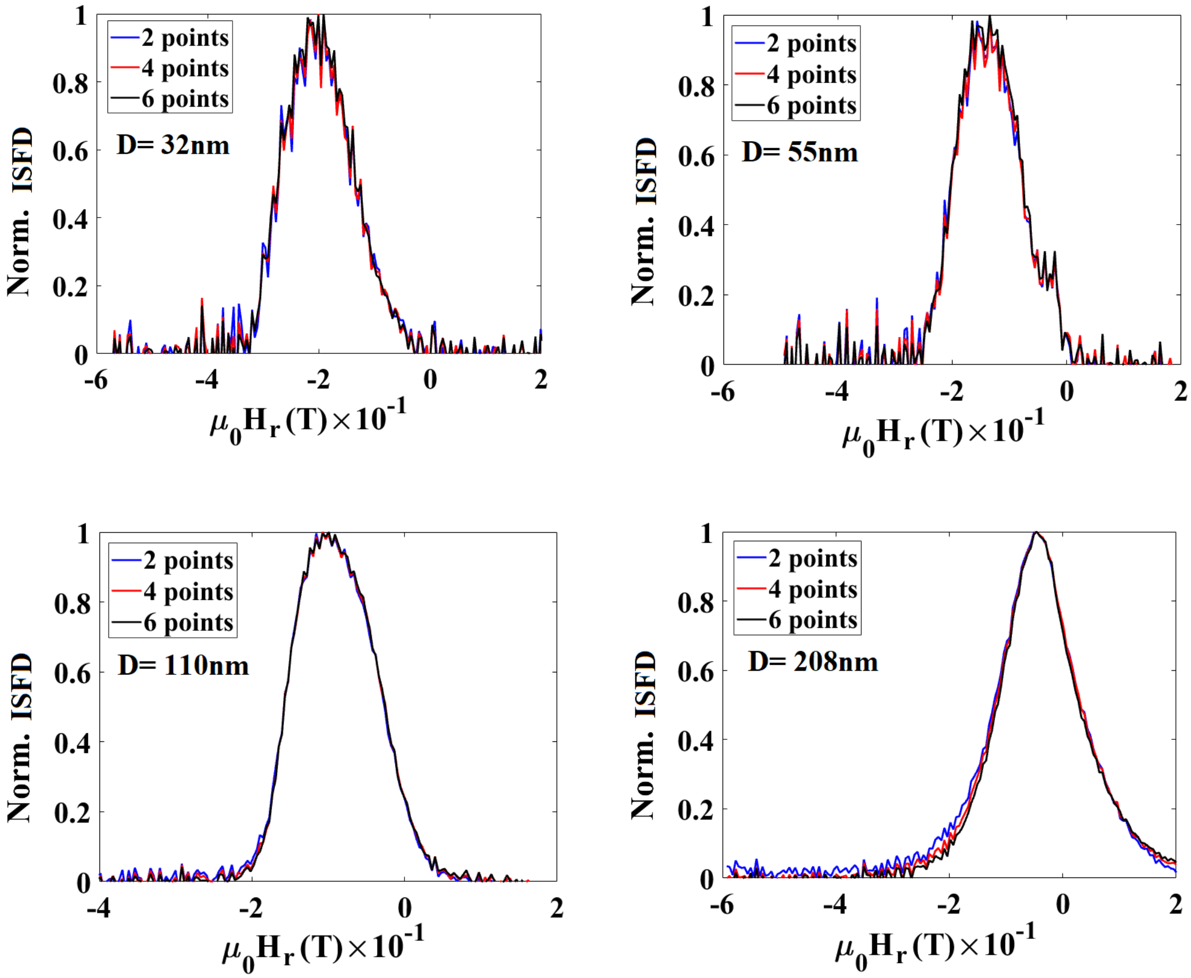

Figure SI-5: Illustrating the number of collected data points on each reversal curve. In all subfigures, "Norm." stands for "Normalized". For MNWs arrays with diameters of 32nm and 55nm, since the porosity is very small, the MNWS to not exert much interaction on each other. This causes more noise in the ISFD data.

Data analysis

We electrodeposited several types of the MNWs inside polycarbonate (PC) templates, and then we performed the FORC measurements on each individual sample. We next used the FORCinel software to extract the magnetic signatures (P_{Hc}, P_{Hu}, ISFD, and BRM) for each individual type of MNWs. Then, we combined the PC templates while the MNWs are embedded inside them. To do this, we simply place the PC templates on top of each other, see Figure SI-6. The results for the combined PC templates were similarly analyzed using FORCinel to extract the P_{Hc}, P_{Hu}, ISFD, and BRM. We called this data the “Exp. data” and plotted them in the main text. Finally, to analyze the capability of each magnetic signatures for quantifying the amount of each type of MNW in a sample, the signatures for the combined samples were fit according to the corresponding signatures of the individual MNWs. The “calibration curve” is the weighted summation of the corresponding signatures of the individual types, see Eq. (1). The fitting quality was evaluated using the root mean square (RMS) error of the difference between the “Exp. data” and “calibration curve”, Eq. (2). The RMS error was minimized to find the optimum weights that give the volume ratio (x) of each type of the MNWs in the combination, Eq. (3).

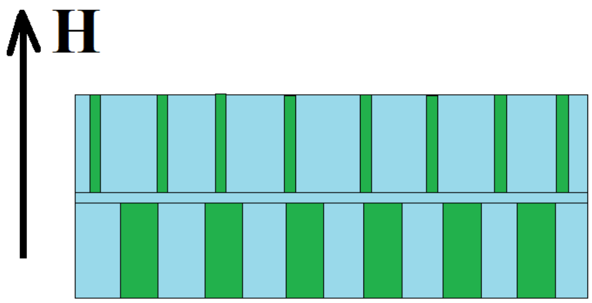

Figure SI-6: A schematic showing the combinations and the measurement direction, black arrow.
